# How Well Do High-Achieving Undergraduate Students Understand School Algebra?

**DOI:** 10.1007/s42330-022-00256-9

**Published:** 2023-02-11

**Authors:** Diarmaid Hyland, Ann O’Shea

**Affiliations:** 1 School of Mathematics and Statistics, University College Dublin, Dublin, Co. Dublin Ireland; 2 Department of Mathematics and Statistics, Maynooth University, Maynooth, Co. Kildare Ireland

**Keywords:** Introductory algebra, Conceptual understanding, Transition to third level, Diagnostic testing, Online assessment, Concept inventory

## Abstract

The aim of this research is to investigate how well high-achieving students entering tertiary-level education in Ireland understand school algebra. As part of a larger project, a 31-item test was developed to assess first-year undergraduate students’ understanding of basic algebraic concepts. The test was administered online to students studying at least one mathematics module at tertiary level and received 327 responses. In this article, we study how the subset of high-achieving undergraduates in our sample performed on the test. The results demonstrated a very high level of understanding among students, as befits their level of study and prior achievement relative to the difficulty of the test. However, one subsection of the test stood out as being disproportionately difficult for these students. The section focused on valid solutions of equations and inequalities. The items in question are described in detail in this article as is the associated data. Our analysis shows that this topic is an area of concern even for high-achieving undergraduates and so deserves further attention. We conclude with a discussion of the implications of this research and details of the larger project.

## Résumé

L’objectif de cette recherche est d’étudier le niveau de compréhension de l’algèbre scolaire d’étudiants performants qui accèdent au niveau tertiaire d’enseignement en Irlande. Dans le cadre d’un projet plus vaste, on a élaboré un test de 31 questions pour évaluer la compréhension des concepts algébriques fondamentaux par les étudiants du premier cycle. On a soumis le test en ligne à des étudiants inscrits à au moins un module de mathématiques au niveau tertiaire et nous avons reçu 327 réponses. Dans cet article, nous examinons parmi notre échantillon les résultats obtenus par le sous-ensemble d’étudiants du premier cycle performants. Par rapport à la difficulté du test, les données indiquent un très haut niveau de compréhension parmi les étudiants, conformément à leur niveau d’étude et leurs résultats antérieurs. Cependant, une sous-section du test s’est avérée plus difficile de façon disproportionnée pour ces étudiants. Cette section portait sur les solutions valides d’équations et d’inéquations. Dans cet article, on décrit en détail les éléments en question ainsi que les données qui leur sont associées. Notre analyse montre que ce sujet est préoccupant, même pour les étudiants de premier cycle performants, et qu’il mérite donc une attention particulière. Nous concluons par un débat sur les implications de cette recherche et les détails associés au projet plus vaste.

## Introduction

This research was undertaken as part of a larger project on the development of an Algebra Concept Inventory (ACI). The ACI is being designed and administered to gather information in order to better support students studying algebra at tertiary level in Ireland (Hyland et al., 2021). Algebra is fundamental to the study of mathematics and has a rich history across all levels in mathematics education research. Algebra is a multifaceted topic, with research focusing on the concepts of expressions (McNeil et al., 2010), variable (Knuth et al., 2005), equivalence (Knuth et al., 2005), fractions (Pantziara & Philippou, 2012), ratio (Lamon, 2012), and word problems (Capraro & Joffrion, 2006; Clement et al., 1981) at primary and secondary level.

Bush and Karp (2013) conducted a thorough literature review relating to algebraic skills of second-level students. They structured the topics of interest into four categories: ratios and proportional relationships; number systems; expressions and equations; and functions. Of particular interest in this paper are items designed to assess students’ understanding of solving equations and inequalities correctly within algebra. Bush and Karp (2013) reported on students’ difficulties with equations, including checking solutions (Perrenet & Wolters, 1994), reversal order error (Abouchedid & Nasser, 2000), symbolic notation (Swafford & Langrall, 2000), combining like terms (Ashlock, 2006), distributive property (Ashlock, 2006), and inverse operations (Ashlock, 2006). Many studies have reported on students’ view of the equals sign as an instruction to carry out a computation (e.g. Knuth et al., 2005) and the problems that this causes them when solving algebraic equations (Stacey & MacGregor, 1997). Harel and Sowder (2005) assert that procedural shortcuts such as ‘cross multiply’, ‘invert and multiply’, and ‘move the decimal point’ are often presented to second-level students as rules with no explanation as to when or why these manipulations are valid, and that this causes students difficulty when solving equations or inequalities. With respect to manipulating equations, the concept of division by zero is poorly understood, as reported by Ball (1990), who discovered fragmented understanding among prospective teachers. O’Connor and Norton (2016) found that students sometimes attempt to use methods suited to solving linear equations in the context of quadratic equations. They also found that even when a quadratic equation was presented in factorised form, students had problems finding solutions because of misconceptions regarding the null factor law (that is, if $ab=0$, then either $a=0$ or $b=0$). In addition, Vaiyavutjamai and Clements (2006) found that students often lacked understanding of the meaning of variables and solutions in the context of quadratic equations. Tsamir et al. (1998) have reported that the most common issue for students solving inequalities is when incorrect parallels are drawn between equations and inequalities, and students may confuse the solution set of an inequality with the solution set of the associated equation (Almog & Llany, 2012).

Studies investigating the understanding of algebra among tertiary-level students exist (e.g. Galbraith & Haines, 2000), but are far less common (Ay, 2017). Tariq (2008) reported that the issues with algebra uncovered at school level persist at tertiary level, making specific reference to difficulties that word problems cause for bioscience students. Similarly, Stewart and Reeder (2017) identified basic algebra errors in cancellation of symbols, evaluating limits, and linear algebra which occur with undergraduate students. Pure mathematics undergraduate students may also struggle with basic concepts. Anderson et al. (1998) report on third-year mathematics students who display an unsatisfactory grasp of topics they covered during their first year of study. They gave students a test containing questions on a range of mathematical material which included an item on algebra. The authors suggest that the mathematics content taught in the first year (which may be considered ‘core material’) is ‘poorly understood or badly remembered’ (Anderson et al., 1998, p. 417).

### Irish Context

The Leaving Certificate is a set of high-stakes summative assessments completed by students at the end of their second-level education in Ireland. The results are used in a points-based system which determines entry to tertiary-level education. Almost all Leaving Certificate students study mathematics; the subject is offered at three levels (Honours, Ordinary, and Foundation). Table 1 shows the possible grades, as well as the percentage of students nationally who were awarded each grade in 2020.

**Table 1:** Leaving Certificate mathematics grades, points, and student achievement in 2020

Leaving Certificate grades, points, and percentage of students who achieved each in 2020								
Higher-level grade	Points	Students awarded each grade (%)	Ordinary-level grade	Points	Students awarded each grade (%)	Foundation-level grade	Points	Students awarded each grade (%)
H1	100	3.1	O1	56	2.7	F1	20	0.4
H2	88	5.5	O2	46	7.6	F2	12	0.9
H3	77	7.8	O3	37	12.2			
H4	66	8.4	O4	28	13.7			
H5	56	6.6	O5	20	11.6			
H6	46	3.7	O6	12	7.9			
H7	37	0.8	+ 25 ‘bonus points’ for achieving a H1–H6 in mathematics					

In 2020, COVID-19-related closures of schools resulted in a change in assessment, with teacher evaluations resulting in calculated grades replacing traditional written examination. The number of students who were enrolled in mathematics for the Leaving Certificate that year was 56,985, while the proportions who were registered at each level were 36% at Higher level; 59% at Ordinary level; and 4.5% at Foundation level (State Examinations Commission, 2020). When compared internationally using PISA scores from 2018, Ireland performs slightly above the mean with respect to mathematics (500 vs. 489). Interestingly, our students outperform the OECD average at attaining Level 2 or higher (84% vs. 76%), but fell below the OECD average for students attaining at least Level 5 (11% vs. 8%) (McKeown et al., 2019). This seems to suggest that, on average, our school system is doing well at creating a minimum level with its students but not doing as well at producing higher achievers.

In Ireland, algebra is introduced to students at primary level and is central to their mathematical learning throughout secondary level. This means that virtually all students study algebra from school entry until they complete secondary school, albeit to different levels. Algebra is one of five strands in the Leaving Certificate curriculum (Department of Education and Skills, 2015). Students who take the Higher level course learn about solving linear, quadratic, and cubic equations, and systems of simultaneous linear equations with three unknowns. They also study the solutions of linear and quadratic inequalities as well as inequalities involving absolute values. The curriculum document specifies that students should be able to use graphical, numerical, and algebraic strategies to solve equations and inequalities.

Many students continue to study algebra at tertiary level; however, here, their algebra education diverges from a common curriculum to better align with their chosen degree. For this reason, we chose to study incoming tertiary-level students. Choosing this cohort means that participants are relatively homogenous with respect to prior learning and can offer common descriptions of their prior achievement—their Leaving Certificate grade.

In the next section, we will detail the achievement of our participants, whom we consider to constitute a subset of high achievers when compared to the overall population data in Table 1. Research on high achievers’ misconceptions in algebra is rare, though Grouws (1996) reported a difference in how high achievers view mathematics (and algebra specifically). An example of the differences in views is described by Grouws (1996), who conducted extensive interviews with students and found that talented students consistently viewed mathematics as a coherent system, whereas non-high-achieving students viewed it in a more fragmented, less connected way.

### Diagnostic Testing

Most first-year undergraduate mathematics modules in Ireland contain students with a wide range of previous mathematical achievement; in particular, many of the students will not have studied mathematics at Higher level in school. Supports for students who are deemed ‘at risk’ of failing, such as extra tutorials and bridging courses, are common (Berry et al., 2015). In order to identify these students, most mathematics departments administer diagnostic tests to their incoming students (Hyland & O’Shea, 2022). There are several purposes for diagnostic testing, but a primary aim is to gather information on the preparedness of incoming students to inform staff (LTSN, 2003). Hyland and O’Shea (2022) conducted an item-by-item analysis of the tasks included on each diagnostic test administered in Ireland, finding that items assessing algebra account for 35% of total test items, which is illustrative of its importance at tertiary level. Furthermore, diagnostic tests are almost exclusively comprised of items with a procedural focus (Hyland & O’Shea, 2022). In contrast, the test used in this study, the ACI, aims to elicit information about students’ understanding of concepts.

### Concept Inventories

A concept inventory is an assessment containing items that focus on conceptual understanding of a given topic, as opposed to factual recall or procedural competence. Concept inventories are well established in STEM education research, beginning in physics in the early 1990s with the Force Concept Inventory (Hestenes et al., 1992), which was based on the Mechanics Diagnostic Test (Halloun & Hestenes, 1985). Hestenes et al. (1992) describe the items on the FCI as requiring a ‘forced choice between Newtonian concepts and commonsense alternatives’, which they label as *misconceptions* (Hestenes et al., 1992, p.142). Though definitions of misconceptions vary within the literature, we use it as explained by Hammer, to be a ‘strongly held, stable cognitive structures, which differs from expert thinking’ (1996, p.1318). Hestenes et al. (1992) state explicitly that the Force Concept Inventory is a probe of belief systems, *not* a test of intelligence. The success of concept inventories in physics has seen them migrate to other STEM subjects including engineering (Wage et al., 2005), chemistry (Dick-Perez et al., 2016), biology (Garvin-Doxas & Klymkowsky, 2008), and mathematics (O’Shea et al., 2016). Concept inventories are typically comprised of multiple choice questions (or items) and are characterised by attempting to assess understanding rather than calculation skills. In a mathematics context, this is analogous to all items requiring *relational understanding*, as opposed to *instrumental understanding*, as described by Skemp (1976). Skemp defined instrumental understanding as ‘rules without reasons’ whereas relational understanding as ‘knowing both what to do and why’ (1976, p. 2).

Hestenes et al. (1992) identifies three potential uses for concept inventories: as placement exams, diagnostic tools, and to evaluate instruction. As diagnostic tools, they have been used extensively to identify misconceptions held by students. This is done primarily through question construction—where the options presented to students consist of the correct solution accompanied by alternatives that address a misconception about the topic at hand. Another tool that can be used to identify misconceptions is a Certainty of Response Index (CRI). The CRI method is used to distinguish between lack of knowledge and misconceptions at both individual and group level. The approach was used by Hasan et al. (1999) on Halloun and Hestenes’ (1985) Mechanics Diagnostic Test, the precursor to the Force Concept Inventory. In essence, the authors (Hasan et al., 1999) attach a CRI to each item to identify how confident the student was in their response: Responding with low confidence, independent of the student’s correctness, is an indicator of a lack of knowledge; high confidence with a correct answer is a justification of the students’ confidence in their answer, but high confidence attached to an incorrect answer may indicate a misconception (Hasan et al., 1999). The same statements vis-á-vis confidence and answer selection can be made at the group level, where the average CRI response of the group is taken into consideration. Engelbrecht et al. (2005) noted when using CRI that it is possible for students to be overconfident in general, or simply to make a mistake especially on a procedural task. However, while the former should be considered in the case of this research, the absence of calculations on the ACI eliminates the possibility of the latter. Beyond concept inventories, CRIs are also used in diagnostic testing generally (e.g. Arslan et al., 2012). The ACI was designed to include a CRI with each question, and the data is used to frame the results.

We believe the fundamental difference in nature between concept inventories and diagnostic tests, which are currently in use in Ireland, justifies the development of the ACI. Its focus on algebra (specifically, conceptual understanding) provides a unique lens through which student understanding can be examined. Furthermore, its capacity to identify misconceptions is a first step toward the identification and subsequent correction of algebra errors at undergraduate level.

The aim of the paper is to describe how well a high-achieving subset of first-year students studying at least one mathematics module at tertiary level in Ireland understand school algebra and to identify areas of particular strength or weakness. The research questions are as follows:

1. How well do high-achieving students (entering university) understand school algebra?
2. Are there particular areas of algebra that present difficulties to these students?

By ‘school algebra’, we mean algebra that is learned at second level in Ireland. Specifically, only content that is studied and assessed at Ordinary level on the Leaving Certificate (see National Council for Curriculum and Assessment, 2021). Answering the research questions will describe the level of understanding students possess in relation to basic algebra during their first year of study at tertiary level in Ireland. Information about specific concepts of algebra that students have difficulty with will be useful to practitioners, enabling the adaption of their approaches in subsequent modules and informing the subsequent stages of our larger research project. In the following sections, we outline the methods of data collection and analysis, present the results of the study, and situate a discussion in the context of the literature.

## Methodology

In this section, we describe aspects of our study before detailing the methods of data collection and analysis used in this study.

### The Algebra Concept Inventory

The Algebra Concept Inventory (ACI) consists of 31 multiple choice questions, each paired with a certainty of response index. It is designed to test incoming tertiary-level students’ understanding of school algebra. As part of the design of the ACI (Hyland et al., 2021), the research team conducted an ‘algebra decomposition’, which targeted six main conceptual areas in the topic of algebra: equality, solutions, variables, expressions, operations, and multiple external representations (MER). This is an acknowledgement of the breadth of the topic and ensures that the ACI is a comprehensive test of students’ understanding of school algebra. The six-factor decomposition has several uses throughout the design stage; one is to gauge how well prospective items combine to form a well-rounded assessment. This is done by mapping each of the items on the test to the decomposition. In total, seven items mapped to Variables and MER, eight to Operations, nine to Expressions, 12 to Solutions, and 13 to Equality, which is indicative of a well-balanced assessment of algebra. All items were multiple choice (three options each) and did not require students to use algorithms or procedures to answer the questions. That is, the items aimed to test students’ underlying relational understanding rather than their instrumental understanding (Skemp 1976) or procedural fluency (Kilpatrick et al., 2001). The items discussed in this paper were designed by the research team, though two were adapted from previous research (Q20—Mason, 2002; Q23—Hyland, 2018).

Each test item was paired with a certainty of response index, meaning participants associated their answer with their level of confidence (the possible responses were as follows: very confident, somewhat confident, not confident). Using certainty of response indices informs the identification of misconceptions. An incorrect response paired with high confidence is indicative of a misconception whereas an incorrect (or correct) response with low associated confidence suggests the participant is guessing. A correct response paired with high confidence shows good understanding of the concept.

### Data Collection

The ACI was administered digitally due to COVID-19 restrictions in place at the time of the rollout and was advertised exclusively through email. The authors forwarded the test to tertiary-level mathematics lecturers in Ireland through various mailing lists, and many of the lecturers forwarded the test to their students. It was open from February 3 to May 5, 2021, during which time it recorded the responses of 327 first-year students. This study received ethical approval from the research ethics committee at the authors’ institution prior to commencement.

### Participants

In total, 327 students from 10 tertiary-level institutions in Ireland responded to the test. A plain language statement preceded the test, and informed consent was received from all students reported in this study. Data was also gathered on the students’ degree programme, as well as the grade that they were awarded on their Leaving Certificate mathematics examination.

The degree programmes most frequently represented in our sample were engineering courses (22%), specialist mathematics (18%), general science (11%), and computing (9%). Students studying a single science subject (e.g. physics, chemistry, or biology) combined for 24% of the respondents, and initial teacher education programmes totalled 7%. The remaining participants (9%) were studying courses in business, technology, philosophy, or psychology, or studying mathematics as part of a Bachelor of Arts programme. Receiving responses from students studying a wide range of subjects is desirable because it illustrates a heterogeneous group of participants with respect to primary interest.

**Fig. 1:**
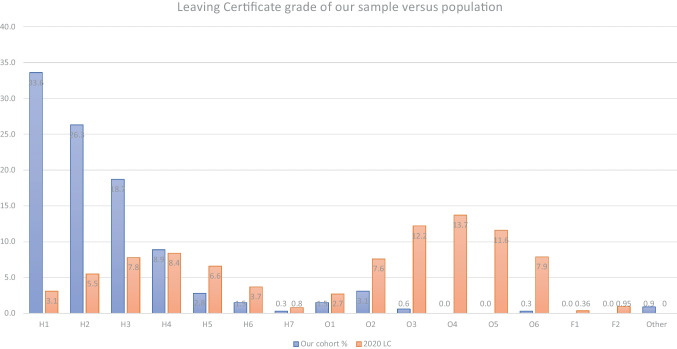
Leaving Certificate grades for our sample (blue) versus those of the population (orange)

As can be seen from Fig. 1, the Leaving Certificate grades of the participants differed significantly from the population of school leavers in that our sample contains an abnormally large group of high-achieving students. Though we received responses from students who studied mathematics at all levels, one-third of our cohort were awarded an H1 grade in their Leaving Certificate (compared to 1.88% of the entire Leaving Certificate cohort) with a further quarter awarded an H2 (compared to 5.17% of the Leaving Certificate cohort).

The disparity between our participants’ achievement on the Leaving Certificate and that of the general population caused us to change the focus of our research from all participants to the subgroup of very high achievers. We include all students who achieved an H1 or H2 in our subgroup. This definition includes 199 participants in our analysis and is equivalent to being at or above the 93^rd^ percentile nationally, which we believe justifies their label of ‘high achieving’. The proportions of students in this group enrolled in the various degree programmes listed earlier were very similar to the overall data: engineering courses (27%), specialist mathematics (26%), a single science subject (19%), computing, (11%), general science (10%), Bachelor of Arts (6%), and initial teacher education (5%).

### Data Analysis

A considerable amount of quantitative data was collected in response to the test. This data consists of the responses to the 31 test items and associated certainty of response indices which were both in multiple choice format. This data was tallied and is presented as text in the results section with associated tables and figures.

## Results

In this section, the results of the study are presented: 327 students from 10 tertiary-level institutions in Ireland completed the ACI. The research questions focused on a high-achieving subgroup (*n* = 199) of the participants, and their understanding of school algebra. Though these students are our focus, we present some results for the entire group followed by a more detailed look at the performance of the high-achieving students. We begin with total score data, before looking at individual item performance. We then focus on a cluster of items with a common underlying theme.

### ACI Scores

**Fig. 2:**
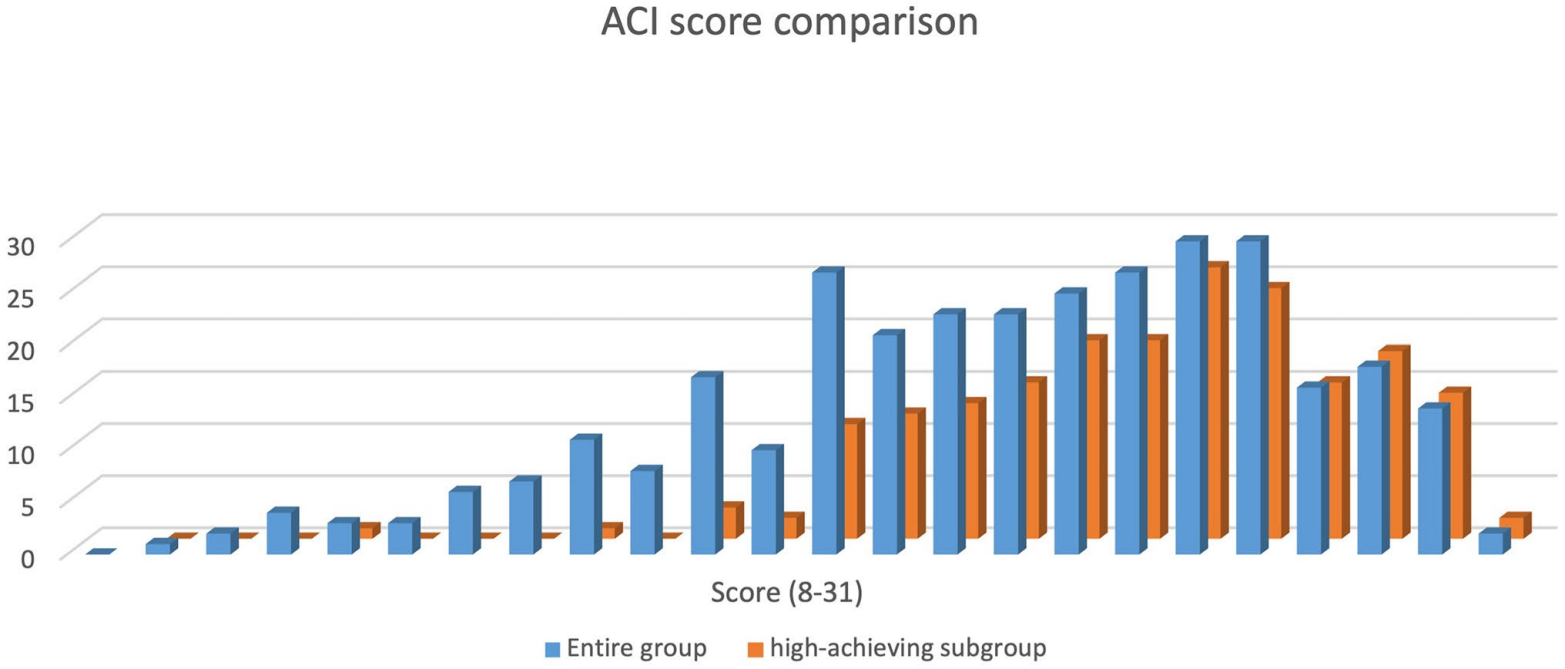
ACI scores of the entire group and the high-achieving subgroup

Given the level of education of our participants and their familiarity with the content, it was not surprising to see such high success on the ACI across the entire group. Two participants successfully answered every question (31 items), and all participants answered at least eight questions correctly on the ACI. For the entire group, the mean is 22.8, median is 23, and mode is 26 and 27, with standard deviation of 4.7. Figure 2 shows the distribution of scores across all participants.

When we look at the high performers as a group (*n* = 199), we see similar levels of success. We notice that both of the students who achieved perfect scores on the ACI belong to this subgroup and that the lowest score of students in this subgroup is 12, and only seven of these students scored less than 20. We also see increases in the mean (25.1) and median (26), and a decrease in the standard deviation (3.3).

### Item Success for ACI

Success on individual items was also investigated for the entire group and our high-achieving subgroup, and are presented together below (Table 2).

**Table 2:**
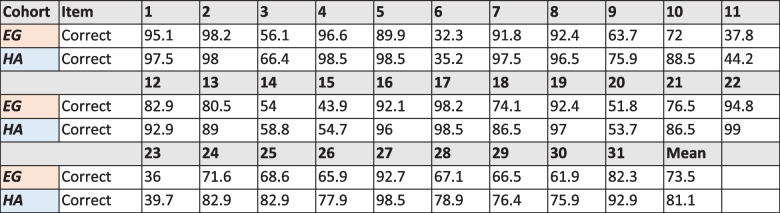
Item success of the entire group (EG) compared to the high-achieving (HA) subgroup

It is noteworthy, though perhaps expected, that the high achievers had greater success on every question than did the entire cohort. Looking at the questions with the lowest success, a cluster of six separate from the others for the entire group and for the high-achieving subgroup (6, 11, 14, 15, 20, and 23) with complete overlap. Even though the H1 and H2 students achieved very high scores on the ACI, each of these questions (6, 11, 14, 15, 20, and 23) had significantly lower proportions of correct answers than the other items. In fact, on average, these six items had a 44.4% success rate which is significantly below the 81.1% average across the 31 items on the ACI.

Upon investigating these items further, it was noticed that questions 11, 15, 20, and 23 all related to the same areas of algebra: the meaning of solutions or valid methods of solving equations or inequalities. These topics (and items) were all classified as belonging to the conceptual area *Solution* on our algebra decomposition. Therefore, these four questions, which are four of the six hardest on the ACI, represent difficulties that even the strongest students face with this topic. The other two items on this list concerned different areas of algebra: Item 6 was on the topic of expressions, and item 14 was a graphical question. We will consider questions 11, 15, 20, and 23 in more detail as these items shared a common theme, namely the solution of equations or inequalities. We will describe each item before discussing the high-achieving students’ responses. Note that the correct answers are given in bold.

#### Item 11 (44.2% Success Among High Achievers)

Consider the linear equation $fx+gy+h=0$, where $f$, $g$, and $h$ are real numbers and $f$ and $g$ are nonzero. Which of the following statements are true about the solutions to the equation, where a solution is a pair $(x,y)$?

- There is exactly one solution to the equation.
- The number of solutions depends on $f$, $g$, and $h$.
- **There are infinitely many solutions to the equation**.

This question asks students to consider the size of a solution set where the solution set is a line in ${R}^{2}.$ This type of solution set is probably unfamiliar to these students, and this may account for the lack of success with this question. Both distractors appealed to a significant proportion of the participants (a, 25%; b, 37%; see Table 3), though not at the confidence level that would indicate the presence of a misconception (we will discuss this issue later). Despite the difficulty it caused our high-achieving subset, no related research has been found on this topic. Its design and inclusion are based on observations by members of the design team from previous work.

#### Item 15 (54.7% Success Among High Achievers)

Examine the student’s solution technique for an inequality shown below and choose the correct feedback.

$$\begin{array}{l}2{x}^{2}-8<0, x\in {\mathbb{R}}\;\;\,\quad{Add\; }8\mathrm{\; to \;both \;sides}\\2{x}^{2}<8\quad\qquad\qquad\;\;\; {\;Divide\; both\; sides\; by\; 2}\\{x}^{2}<4 \qquad\qquad\qquad\;{\;Take\; the\; square\; root\; of\; both\; sides}\\x<2 \quad\qquad\qquad\quad\;\;\;\, {{Student}^{\prime}s \;solution\; to\; the\; inequality}\end{array}$$

- $x<2$ is the correct solution for this inequality.
- $x<2$ is incorrect/incomplete because “Divide both sides by $2$” is not a valid operation in this case.
- $\mathbf{{\varvec{x}}<2}$  **is incorrect/incomplete because ‘Take the square root of both sides’ is not a valid operation in this case**.

Item 15 assesses students’ understanding of valid manipulations of inequalities and focuses on an operation, which has different ramifications in the solutions of equations and inequalities. In this question, students need to realise that taking the square root in the final step is not valid; that operation leads to a solution set of $\left\{x|x<2\right\}$, which is clearly not correct (for example, notice that $-3<2$ but $x=-3$ is not a solution to the inequality). The actual solution set is $\{x|-2<x<2\}$. Through participant responses, we can surmise that option b is a non-functional distractor that requires revision. This term describes a distractor which appeals to less than 5% of participants (Sajjad et al., 2020). We also identified option a as a potential misconception (Table 3). Option a was designed to appeal to students who consider solving inequalities to be ‘the same’ as solving equations, previously discussed by Tsamir and Bazzini (2004).

#### Item 20 (53.7% Success Among High Achievers)

Examine the following solution and explanation provided by a student. Choose the correct student response.

$$\begin{aligned}{x}^{2}&=4x\; \quad Divide\; both\; sides\; by\; x\\x&=4\\\mathrm{Answer}: x&=4\end{aligned}$$

- Yes, this is correct because when you substitute 4 for *x*, you get 16 = 16.
- **No, this is not correct**. $\mathbf{{\varvec{x}}=4}$  **is a solution to the equation** $\mathbf{{{\varvec{x}}}^{2}=4{\varvec{x}}}$, **but dividing both sides by** ***x*** **is not a valid** **manipulation in this instance**.
- Yes, the student carried out a valid manipulation (dividing both sides by *x*) and obtained all values of *x* that satisfy the equation ${x}^{2}=4x$.

This item is similar to item 15, as once again students are asked to provide feedback on a fictional student’s answer. In this case, we wanted to check whether students spotted the issues that arise when dividing both sides of an equation by the variable *x*. In particular, we wanted to see if they realised that $x=0$ was also a valid solution to the equation. The incorrect options were designed to appeal to participants who do not understand the issue with division by *x* (c), or those who assume only one solution exists (a). Option c was informed by the difficulties identified by O’Connor and Norton (2016), and by Ball (1990) who reported students displaying a poor understanding of division by zero. Option a was chosen because of a misconception observed anecdotally within the larger design team, though for which no prior research was found.

#### Item 23 (39.7% Success Among High Achievers)

Equation 1, below, has $x=1$ as a solution. Examine the solution technique and choose the most accurate answer﻿.

1 $$x.a+a=0, x\in {\mathbb{R}}$$

$$\begin{aligned}&a(x+1)=0\!\quad\mathrm{Step}\;1:\;\mathrm{Factorise}\\& x+1=0\;\qquad\,{\mathrm {Step}}\;2:\;\mathrm{Divide}\;\mathrm{both}\;\mathrm{sides}\;\mathrm{by}\;a\\& x=-1\qquad\qquad\qquad\quad\;\mathrm{Step}\;3:\;\mathrm{Subtract}\;1\;\mathrm{from}\;\mathrm{both}\;\mathrm{sides}\\& x=-1\qquad\qquad\qquad\quad\;\mathrm{Solution}\end{aligned}$$

- All of the steps of the solution technique are correct.
- Step 1 is not valid for some values of $a$.
- **Step 2 is not valid for some values of** ${\varvec{a}}$.

The item is quite similar to item 20, although it could be considered a more difficult question based on the reduced success of participants. Again, we see that option b is a non-functional distractor, and that the majority of students did not consider the case $a=0.$ As with item 15, the item is based on observations from previous work (Hyland, 2018) and elsewhere. As with 20 (c) above, the design of 23 (a) is informed by Ball (1990) and Vaiyavutjamai and Clements (2006).

We see that items 15, 20, and 23 highlight issues that arise when students attempt to solve equations and inequalities especially in relation to which operations are valid. These are common mistakes that students make, but one might expect the high-achieving students to be able to spot the errors in the arguments given. This, combined with their confidence in their answers, points to a ‘blind spot’ or misconception, which we will now discuss.

### Misconceptions

In the previous section, we justified the inclusion of a certainty of response index (CRI) in the ACI. CRIs have been used with concept inventories previously (Hasan et al., 1999), and can be used to identify the presence of misconceptions as follows: responding with low confidence (independent of the student’s correctness) is an indicator of lack of knowledge. High confidence with a correct answer is a justification of the students’ confidence in their answer, but high confidence attached to an incorrect answer indicates a misconception. The same can be said at the group level, where the average CRI response of the group is taken into consideration.

In the table below, we present the high-achieving students’ responses to each of the items, broken down by the option chosen and the associated confidence level. The correct responses are highlighted in bold font, and colour is used to convey information about confidence of response indices: high confidence in the correct response (yellow); high confidence in an incorrect response (or a misconception—red); and guesses (green) are included. By examining Table 3 more closely, we can make statements about the presence of misconceptions and the performance of our distractors.

**Table 3:**
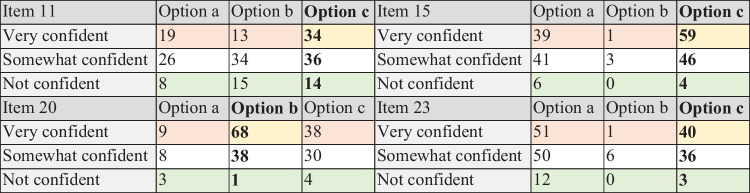
A breakdown of high achievers’ responses to the four items of interest

The Options emphasised in bold are the correct responses to the Item in each case. This is noted in the text directly above the preferred location of the table, and is consistent with the items which appear in text

All student responses, broken down by level of confidence, are contained within Table 3. However, we can focus on the coloured tiles to glean information about guessing (green), good understanding (yellow), and misconceptions (red). Beginning with misconceptions, we are drawn to the following responses: item 15 a; item 20 c; and item 23 a. All three of these responses were chosen by more than 10% of participants with a high degree of confidence which, according to Hasan et al. (1999), signals the presence of misconceptions. We also note the high numbers of students who chose those options with a reasonable level of confidence. Though these students lack the entrenched belief that is typically associated with a misconception, they are drawn to these responses in sufficient numbers to support an argument that the distractors represent a pattern of misunderstanding at the population level.

The responses to items 20 and 23 indicate misconceptions concerning the solution set of an equation of the form $f\left(x\right)g\left(x\right)=0$ and where $f\left(x\right)=x$ or $f\left(x\right)$ is constant. In these situations, many students seem to only consider solutions arising from $g\left(x\right)=0$ and ignore the possibility that $f\left(x\right)$ could be zero. The misconception identified in item 15 also involves students ignoring some elements of the solution set. In that case, they apply an operation to both sides of the inequality without considering if that is valid. Finally, we would like to briefly discuss the performance of the distractors on these items. In order for distractors to be considered ‘functional’, they need to elicit at least 5% of the participants (Sajjad et al., 2020). By this metric, item 15 b and item 23 b should be revised.

## Discussion

This research was undertaken as part of a larger project investigating first-year undergraduate students’ understanding of introductory algebra. Though issues with algebra around the transition to tertiary level are well-documented, studies often focus on procedural fluency as opposed to understanding. In contrast, our test instrument aimed to gain information about students’ understanding of algebraic concepts rather than their procedural fluency in this area. Our data shows a high level of success on the test overall, and so we have evidence that, on the whole, high-achieving first-year students enter university with a good understanding of algebra. However, we have identified *Solutions: What is valid versus invalid* as one concept that causes many students difficulty. When we look at specific errors on our cluster of items, we see a significant number of students drawing incorrect parallels between solving inequalities and Eqs. (15 a—as reported by Tsamir et al., 1998) and displaying a poor understanding of division by zero and the use of the null factor law (20 a/c and 23 a—as reported by Ball (1990) and Vaiyavutjamai and Clements (2006)). Each of these responses meets the criteria identified by Hasan et al. (1999) to be labelled as misconceptions.

We believe this finding is strengthened when the ability of the cohort under investigation is considered. Using Leaving Certificate mathematics grade as a measure of ability, the subgroup of participants on whom we focused (*n* = 199) can be considered high achieving relative to the population who completed the examination. Specifically, these participants achieved at the 93^rd^ percentile or higher of the school leaving population. It might be assumed that such students would not have any difficulty with basic algebra, and indeed, the lack of research on the algebraic knowledge of these types of students may be a result of this view. However, our study has shown that these students do have difficulty with some aspects of solving equations and inequalities and in particular with taking account of all possible cases.

Though Tariq (2008) and others (Booth et al., 2014; Stewart & Reeder, 2017) have reported the existence of errors in school algebra persisting into tertiary level, these issues may not be immediately obvious to lecturing staff. Questions assessing conceptual understanding on mathematics diagnostic tests at tertiary level in Ireland occur very rarely (Hyland & O’Shea, 2022), so practitioners might assume the only difficulties present in their cohort are the ones identified through the diagnostic test. In addition, faculty members might well assume that students who achieve extremely high grades in end-of-school examinations do not need to review any aspects of basic algebra at the beginning of their undergraduate programmes. Our study has highlighted some problems that even very well-prepared undergraduates encounter, and we presume that these issues are even more problematic for the general population. Therefore, we believe that our study throws light on important gaps in student knowledge.

Whether issues are identified upon entry to university or not, the solution to shortcomings in students’ conceptual understanding is not simple. McGowen (2017) noted that problems such as those highlighted in our study are sometimes acknowledged by lecturers but are often not addressed. Furthermore, Booth et al. (2014) explain that typical instruction alone is unlikely to correct such misconceptions, and that specific interventions may be required. They suggest the use of targeted teaching materials with an active learning focus. Indeed, following this advice, we aim to develop a range of teaching materials to help students with concepts within algebra identified during the research. This process has already begun with the design of a series of guided inquiry tutorials to develop understanding of the concept of solution within algebra; the design process was guided by the findings of this study. These materials will be trialled and improved on locally before being made available to all interested practitioners.

## Limitations of the Study

There are aspects of the study that should be taken into account when considering the results. We would like to remind the reader that the results were gathered using an online survey, which presented several challenges. First, it has been reported that collecting data in an online setting has several differences compared to in-person testing (Latkovikj & Popovska, 2020; Szolnoki & Hoffmann, 2013). Some of the reported differences concern response rates and biassed samples. Thankfully, our colleagues were very supportive to our cause, and we received a tremendous response from students. Our sample is not ideal; however, it contains a significant bias toward high-achieving students. This result is disappointing for our larger project, but less so for this study because it gave us an opportunity to study an area of perceived weakness.

The timing of the survey also bears mentioning. As stated previously, advertising a survey during the second semester of study was not ideal. Feedback from lecturers indicated that such a test is more easily shared with students at the start of the academic year. We acknowledge the shortcomings in the timing of data collection and the bias in the sample toward higher-achieving students. We have taken these, and other issues, into account for our larger project, and are recording additional responses at the time of writing this paper.
